# Efficacy and safety of oral gonadotropin-releasing hormone antagonists in moderate-to-severe endometriosis-associated pain: a systematic review and network meta-analysis

**DOI:** 10.1007/s00404-022-06862-0

**Published:** 2023-01-19

**Authors:** Lingli Xin, Yinghao Ma, Mei Ye, Ling Chen, Fuzhou Liu, Qingxiang Hou

**Affiliations:** 1 Department of Obstetrics and Gynecology, PLA Rocket Force Characteristic Medical Center, Xinjiekou Outer Street 16#, Xicheng District, Beijing, 100088 China; 2 Department of Quality Management, PLA Rocket Force Characteristic Medical Center, Xinjiekou Outer Street 16#, Xicheng District, Beijing, 100088 China; 3 Department of Orthopaedics, PLA Rocket Force Characteristic Medical Center, Xinjiekou Outer Street 16#, Xicheng District, Beijing, 100088 China

**Keywords:** Endometriosis, Pain, Oral GnRH antagonists, Efficiency, Safety

## Abstract

**Purpose:**

The aim of this NMA is to comprehensively analyze evidence of oral GnRH antagonist in the treatment of moderate-to-severe endometriosis-associated pain.

**Methods:**

Literature searching was performed to select eligible studies published prior to April 2022 in PubMed, Cochrane, Embase and Web of Science. Randomized controlled trials involving patients who suffered from moderate-to-severe endometriosis-associated pain and treated with oral nonpeptide GnRH antagonists or placebo were included.

**Results:**

Elagolix 400 mg and ASP1707 15 mg were most efficient in reducing pelvic pain, dysmenorrhea and dyspareunia. Relugolix 40 mg was best in reducing the analgesics use. The rates of any TEAEs and TEAEs-related discontinuation were highest in relugolix 40 mg and elagolix 250 mg, respectively, while rates of hot flush and headache were highest in relugolix 40 mg and elagolix 150 mg. Significantly decreased spinal BMD was observed in elagolix 250 mg.

**Conclusion:**

Oral GnRH antagonists were effective in endometriosis-associated pain in 12w, and most of the efficiency and safety outcomes were expressed in a dose-dependent manner, but linzagolix 75 mg was an exception.

## What does this study add to the clinical work

**Table:** 

Endometriosis significantly debilitates psychological well-being of patients and lacks of effective treatment. This review evaluates the efficacy and safety of a new treatment as oral GnRH antagonists in treating endometriosis-associated pain and is beneficial in making of clinical treatment strategies.	

## Background

Endometriosis, an estrogen-dependent inflammatory disease, affects 6–10% women of reproductive age [1] and accounts for 50–60% pelvic pain and up to 50% infertility [2]. Endometriosis-associated pain, which refers to dysmenorrhea, non-menstrual pelvic pain and dyspareunia, significantly debilitates psychological well-being of patients and brings heavy financial burden [3, 4]. First-line drug therapy for endometriosis-associated pain includes nonsteroidal anti-inflammatory drugs (NSAIDs) combined with oral contraceptives (COCs) and progestogens. However, NSAIDs are often ineffective and cause treatment-associated adverse effects [5], while COCs and progestogens are prone to cause bothersome side effects, such as weight gain, mood swings, and irregular uterine bleeding, which lead to drug interruption. Moreover, 25–33% patients are primarily resistant to COCs and progestogens [6, 7]. For the second-line drug therapy, injectable depot formulations of gonadotropin-releasing hormone (GnRH) agonists are effective in managing endometriosis-associated pain. Nevertheless, flareup effects and hypoestrogenic adverse events relating to complete estrogen suppression limit the long-term use of injectable GnRH agonists [8].

Oral GnRH antagonists are oral short-acting treatments for endometriosis-associated pain. They inhibit the secretion of estrogen in a dose-dependent manner without flareup effects, and rapid reverse of estrogen-suppression effects can be achieved shortly after drug withdrawn. Thus, it is convenient to tailor dosage to balance efficacy and safety [9]. Currently, oral GnRH antagonists, including elagolix and relugolix, have been approved for endometriosis by FDA [10] (https://www.contemporaryobgyn.net/view/fda-approves-myfembree-for-endometriosis-pain), while treating EAP with Linzagolix and ASP1707 has being evaluated in ongoing clinical trials [11, 12]. However, there is lack of comprehensive comparison on efficiency and safety of different oral GnRH antagonists.

In the present study, we conducted systematic review and network meta-analysis (NMA) to evaluate the efficacy and safety of oral GnRH antagonists in treating endometriosis-associated pain.

## Methods

This study was conducted according to guidelines of Preferred Reporting Items for Systematic Reviews Incorporating Network Meta-analyses of Health Care Interventions with minor modification [13].

### Literature searching

Literature searching was performed to select eligible studies published prior to April 2022 in the following electronic databases: PubMed, Cochrane, Embase and Web of Science. The following combined relevant Medical Subject Heading terms and keywords were used: “elagolix” [All Fields] and “endometriosis” [All Fields], “relugolix” [All Fields] and “endometriosis” [All Fields], “linzagolix” [All Fields] and “endometriosis” [All Fields], “ASP1707” [All Fields] and “endometriosis” [All Fields], and “opigolix” [All Fields] and “endometriosis” [All Fields]. The searching was confined to English language and human studies. Following the searching, duplicate studies were removed by Endnote X7 for windows. The remaining studies were manually screened to identify additional potential studies by two independent authors (Q.X.H. and L.L.X.).

### Inclusion and exclusion criteria

The candidate studies should satisfy the following inclusion criteria: 1. randomized control studies (RCT); 2. studies involving patients suffering from moderate-to-severe endometriosis-associated pain; 3. studies comparing placebo with oral non-peptide GnRH antagonists without addback; 4. human studies published in English; 5. studies reporting any of the following outcomes for 12 weeks: numeric rating score (NRS) of pelvic pain, modified Biberoglu and Behrman (M-B&B) score of dysmenorrhea, M-B&B score of dyspareunia, percentage of days using analgesics, rate of any grade treatment-emergent adverse effects (TEAEs), rate of TEAEs leading to treatment discontinuation, rate of hot flush, rate of headache, spinal and femoral bone mineral density (BMD); and 6. available full text. Studies met following criteria were excluded: 1. review studies, comments, letters, meta-analysis; 2. studies involving peptide GnRH antagonists; 3. treatment with oral non-peptide GnRH antagonists and other pharmaceuticals.

### Data extraction

The data extraction was performed by two independent authors (Q.X.H. and L.L.X.). The following information was extracted: the name of first author, year of publication, country, study design, sample size, age of the patients, treatment arms, duration of follow-up, pain-related outcomes, and safety-related outcomes. For pain-related outcomes, results of change of NRS of pelvic pain, M-B&B score of dysmenorrhea, M-B&B score of dyspareunia, and use of analgesics were collected. For safety-related outcomes, we extracted data of rate of any TEAEs, treatment discontinuation led by TEAE, hot flush, headache, percentage change of spinal and femoral BMD from baseline. If there were more than one study from one cohort with identical outcomes, the more comprehensive study would be included. When the complete data for quantitative synthesis was unavailable, we turn to the correspondence author for full data by email.

### Quality assessment

The quality of eligible studies was assessed by the Cochrane Risk of Bias tool. There are seven components included in the qualification, consisting of random sequence generation, allocation concealment, blinding of participants and personnel, blinding of outcome assessment, incomplete outcome, selective reporting and other biases. In each component, the judgment is categorized as low risk of bias, unclear risk of bias or high risk of bias [14].

### Statistics analysis

NMA was implemented with R software version 4.1.0 for windows. Heterogeneity across studies was evaluated with *P* value and* I*^2^; *P* value > 0.1 and *I*^2^ < 50% indicated low heterogeneity and a fixed-effects model was applied; *P* value < 0.1 and *I*^2^ > 50% indicated significant heterogeneity and a random-effects model was applied. Polled continuous variables were expressed as mean difference (MD) with 95% confidence interval (CI), and pooled dichotomous variables were expressed as relative ratio (RR) with 95% CI. When the 95% CI did not include 0 for MD and 1 for RR, it was considered significantly different. The efficacy of treatments was ranked according to *P* score. A larger *P* score indicated worse pain-related outcomes, higher incidence of adverse effects or higher BMD.

## Results

### Literature searching results

**Fig. 1:**
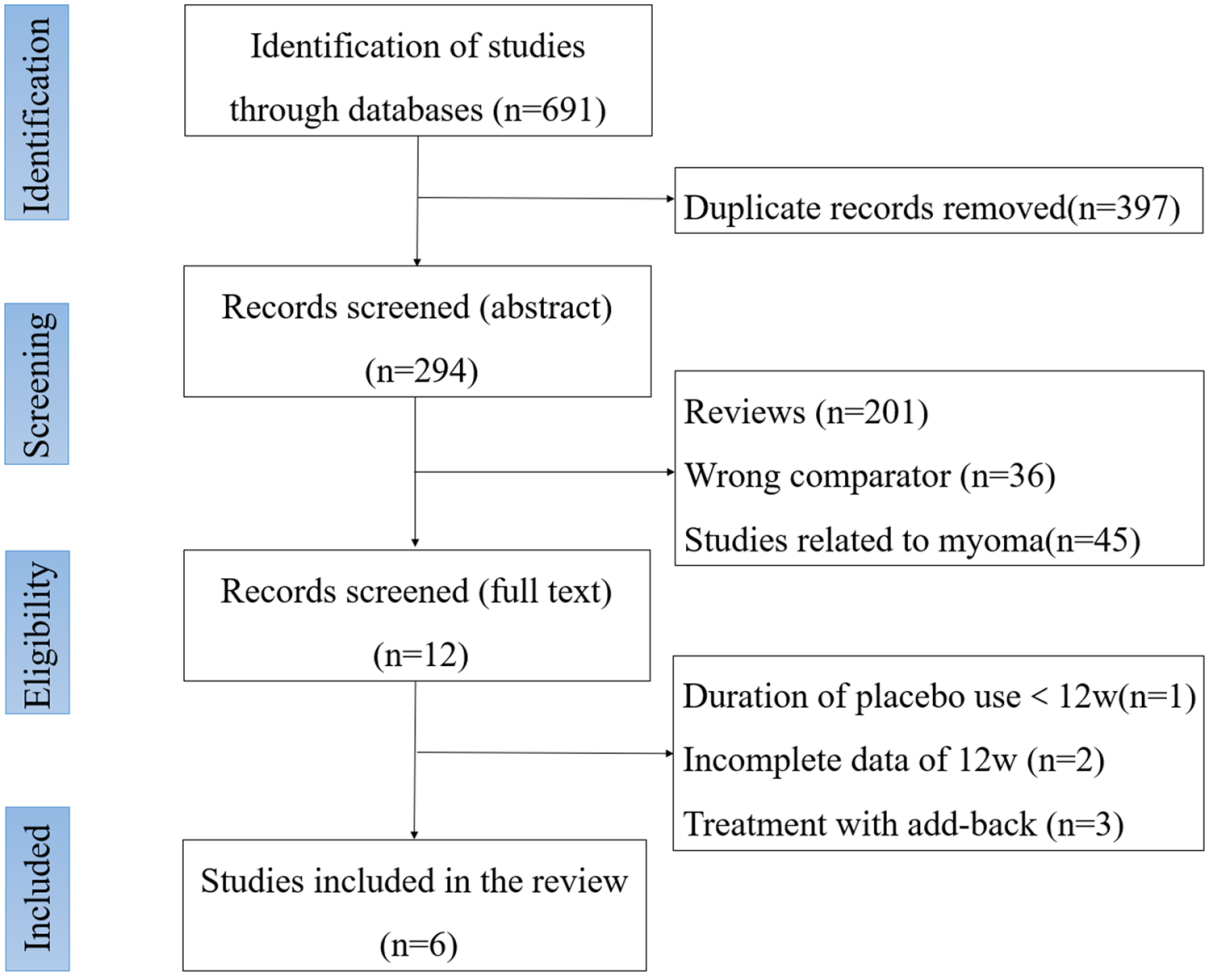
Flowchart of literature searching

A total of 292 studies were identified, and 6 eligible studies were included for subsequent NMA [12, 15–19]. Figure 1 shows the flowchart of study identification.

### Summarized characteristics of eligible studies

A total of 2732 patients in 26 cohorts with moderate-to-severe endometriosis-associated pain were included in our analysis. Of the 6 eligible studies, 3 compared varying doses of elagolix (150 mg and 250 mg or 400 mg) with placebo [15, 17, 19], 1 compared varying doses of ASP1707 (3 mg, 5 mg, 10 mg, 15 mg) with placebo [14], 1 compared varying doses of relugolix (10 mg, 20 mg, 40 mg) with placebo[16], and 1 compared varying doses of linzagolix (50 mg, 75 mg, 100 mg, 200 mg) with placebo [18]. Table 1 shows the summarized characteristics of the 6 eligible studies.

**Table 1:** Summary characteristics of the included studies

Author, year	Country	Study design	Sample size	Treatment arms	Age	Follow-up (weeks)	Inclusion criteria	Exclusion criteria	Outcomes
D’Hooghe 2019 [12]	Europe	Phase II, multicenter, double-blind RCT	443	ASP1707 3 mg	34.7 (22–45)	24w	Women aged 18–45 years with moderate-to severe endometriosis associated dysmenorrhea and non-menstrual pelvic pain, a surgically confirmed diagnosis of endometriosis, and a confirmed regular menstrual cycle of 24–35 days	1. Treatments that alter gynecological endocrinology;	① ② ③ ⑦ ⑧
				ASP1707 5 mg	33.3 (19–45)			2. Surgery for endometriosis within 4 weeks of study initiation;	
	Japan			ASP1707 10 mg	34.2 (20–45)			3. The presence of pelvic or gynecological abnormalities	
				ASP1707 15 mg	33.7 (18–45)				
				Leuprorelin acetate	33.1 (19–45)				
				placebo	33.5 (18–45)				
Diamond 2014 [15]	USA	Multicenter, double-blind	102	Elagolix 150 mg	30.9 (7.1)	24w	Women aged 18 to 49 years, with diagnosis of endometriosis by laparoscopic visualization within 8 years of screening. Patients had a total CPSSS ≥ 6 at screening and scores of at least	1. Patients who administered a GnRH agonist, a GnRH antagonist, or danazol within 6 months of screening, depot medroxyprogesterone acetate within 3 months of screening, or had used hormonal contraception or other hormonal therapy within 1 month of screening	① ④ ⑥ ⑧ ⑨ ⑩
				Elagolix 250 mg				2. Patients who had a history of unresponsiveness to GnRH agonist or antagonist therapy or if they had surgical treatment for endometriosis within 1 month of the start of screening	
				Placebo	31.0 (7.2)		Moderate (≥ 2) for dysmenorrhea and at least mild (≥ 1) for non-menstrual pelvic pain at baseline		
					31.2 (7.2)				
Osuga 2021 [16]	Japan	Phase 2, multicenter, double-blind RCT	405	Relugolix 10 mg	35.3 (6.2)	12w	Women aged ≥ 20 years of age, regular menstrual cycles (25–38 days), a diagnosis of endometriosis in the previous 5 years and dysmenorrhea and pelvic pain due to endometriosis, either one or both of which were at least moderate as determined by the investigator using the B&B scale	1. Measurable UF with the longest diameter ≥ 3 cm;	② ③ ④ ⑤ ⑥ ⑦ ⑧
				Relugolix 20 mg	35.1 (6.8)			2. Lower abdominal pain due to irritable bowel syndrome or severe interstitial cystitis	
				Relugolix 40 mg	35.6 (6.0)			3. Thyroid dysfunction, pelvic inflammatory disease, serious cardiovascular hepatic, renal, or hematologic disorders;	
				Leuprorelin	36.1 (6.1)			4. A positive Papanicolaou smear test result;	
				Placebo	35.7 (6.1)			5. A history of hysterectomy or bilateral oophorectomy	
Taylor 2017 [17]	USA	Phase 3, multicenter, double-blind RCT	1285	Elagolix 150 mg	32 (18–49)	24w	18 and 49 years who had received a surgical diagnosis of endometriosis in the previous 10 years and who had moderate or severe endometriosis-associated pain	1. A z score of less than − 1.5 for bone mineral density at the lumbar spine, femoral neck, or total hip at screening;	①→③
	Canada			Elagolix 400 mg (200 mg, twice daily)	31.4 (18–49)			2. Clinically significant gynecologic conditions or chronic pain conditions unrelated to endometriosis	
				Placebo	32.4 (18–47)				
Donnez 2020 [18]	USA	Phase 2b, multicenter, double-blind, RCT	323	Linzagolix 50 mg	30.9 (6.0)	24w	Premenopausal women aged between 18 and 45 years, with a confirmed surgical diagnosis of endometriosis in the previous 10 years and currently experiencing moderate-to-severe EAP	1. Chronic pelvic pain was not caused by endometriosis;	① ⑤ ⑦ ⑧
	Europe			Linzagolix 75 mg	31.6(6.5)			2. Liver enzyme anomalies, osteoporosis, or other metabolic bone disease	
				Linzagolix 100 mg					
				Linzagolix 200 mg	33.0 (5.8)			3. Patients who taken oral contraceptives, GnRH analogues, or systemic glucocorticoids	
				Placebo	30.9 (6.0)				
					32.4 (5.8)				
Ács 2015 [19]	USA	Phase 2, multicenter double-blind, RCT	174	Elagolix 150 mg	18–45	24w	1. Women aged 18–45 years, with laparoscopically confirmed endometriosis within 60 weeks of screening, and a total CPSSS ≥ 6 with a score of ≥ 2 for dysmenorrhea and a score of ≥ 1 for NMPP	1. Patients who administered a GnRH agonist or antagonist, or danazol within 6 months of screening, depot medroxyprogesterone acetate within 3 months of screening, hormonal contraception or other hormonal therapy within 1 month of screening	④ ⑥ ⑧ ⑨ ⑩
				Elagolix 250 mg				2. A history of unresponsiveness to GnRH agonist or antagonist treatment	
				Leuprorelin					
				Placebo					

① Change in numeric rating score (NRS) of pelvic pain from baseline; ② Change in modified Biberoglu and Behrman (M-B&B) score of dysmenorrhea from baseline; ③ Change in modified Biberoglu and Behrman (M-B&B) score of dyspareunia from baseline; ④ Change in percentage of days using analgesics from baseline; ⑤ Rate of any treatment-emergent adverse effects (TEAEs); ⑥ Rate of TEAEs leading to study drug discontinuation; ⑦ Rate of hot flush; ⑧ Rate of headache; ⑨ Percentage change in spinal bone mineral density (BMD) from baseline; ⑩ Percentage change in femoral BMD from baseline

### Quality assessment results

**Fig. 2:**
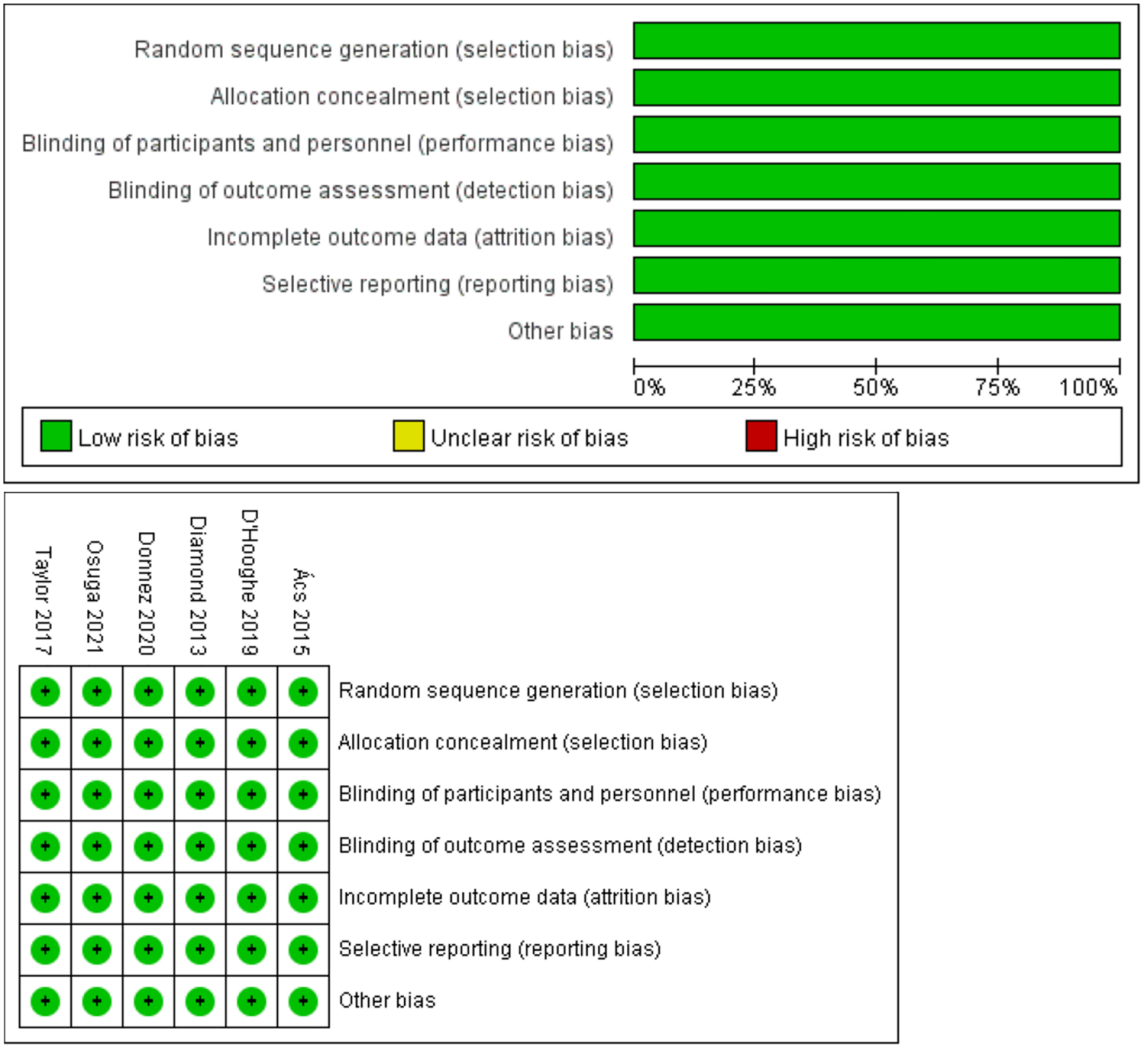
Risk of bias assessment

All the 6 included studies were double-blind RCT with randomization, and allocation concealment and blinding were well implemented. There were no incomplete outcome, selective reporting and other biases in the 6 studies. The risk of bias was assessed as low risk (Fig. 2).

### Outcomes

#### Pain-related outcomes

**Fig. 3:**
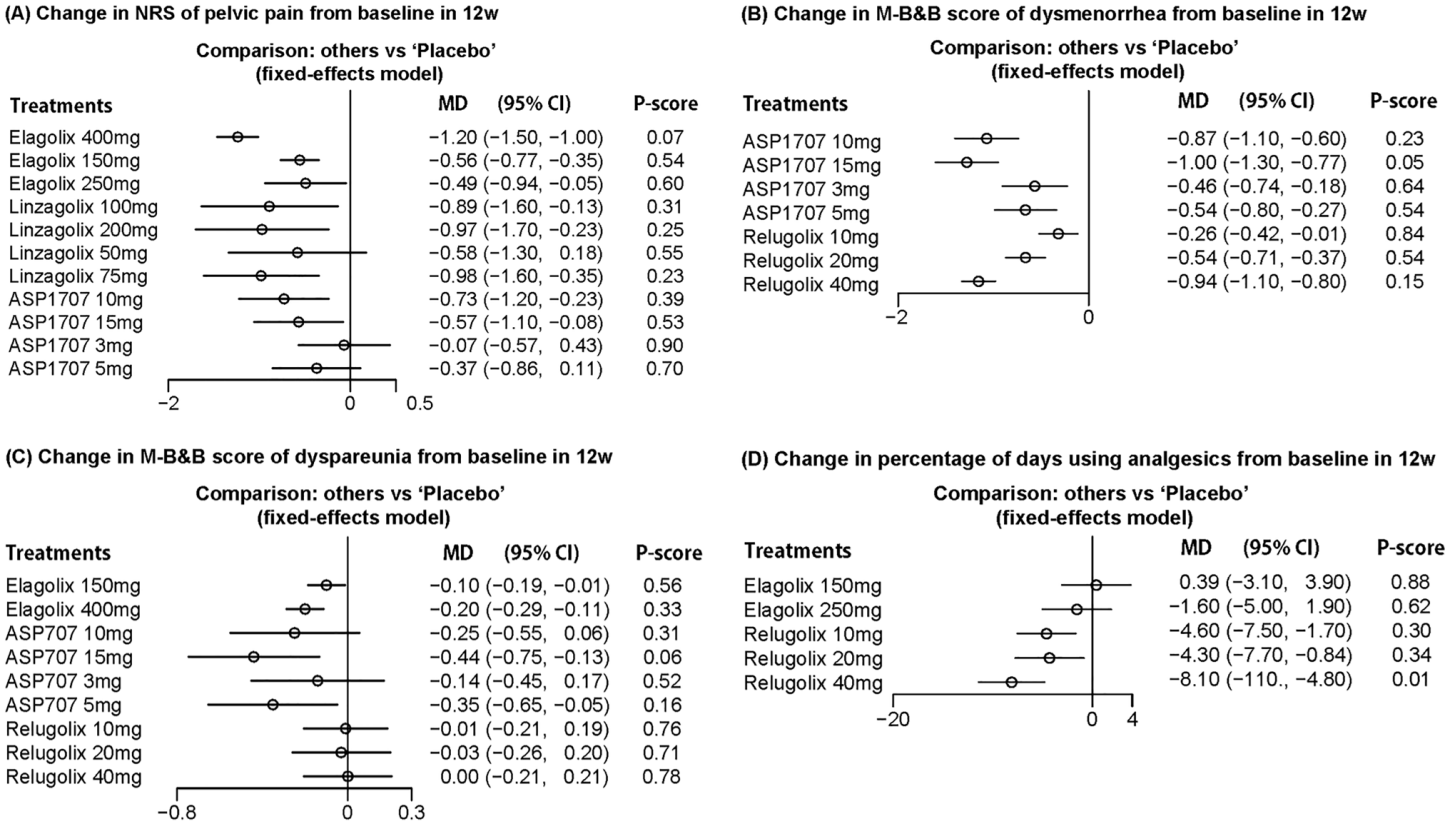
Assessment of pain related outcomes

Four studies reported the change of NRS of overall pelvic pain from baseline in 19 cohorts with a total of 2150 women. Comparing with placebo, elagolix 150 mg, elagolix 250 mg, elagolix 400 mg, linzagolix 75 mg, linzagolix 100 mg, linzagolix 200 mg, ASP1707 10 mg, and ASP1707 15 mg significantly reduced NRS of pelvic pain. No significant difference was found in comparisons between linzagolix 50 mg or ASP1707 3 mg and placebo. The rank of efficacy (from best to worst) was: elagolix 400 mg (0.07), linzagolix 75 mg (0.23), linzagolix 200 mg (0.25), ASP1707 10 mg (0.39), ASP1707 15 mg (0.53), elagolix 150 mg (0.54), linzagolix 50 mg (0.55), elagolix 250 mg (0.60), ASP1707 5 mg (0.70), and ASP1707 15 mg (0.90) (Fig. 3A).

For the change of M-B&B score of dysmenorrhea, 2 studies including 9 cohorts with a total of 846 women were involved. Comparing with placebo, significant reduction of M-B&B score of dysmenorrhea was achieved in all of the treatments. The rank (from best to worst) was: ASP1707 15 mg (0.05), relugolix 40 mg (0.15), ASP170710mg (0.23), ASP1707 10 mg (0.54), relugolix 20 mg (0.54), relugolix 10mg (0.21), ASP1707 30 mg (0.64), and relugolix 20 mg (0.84) (Fig. 3B).

To analyze change of M-B&B score of dyspareunia from baseline, 3 studies with 15 cohorts and 1673 women were included in our NMA. Our results showed that the efficacies of ASP1707 15 mg, ASP1707 5 mg, elagolix 400 mg, and elagolix 100 mg were superior to placebo. Surprisingly, the change of M-B&B score in patients receiving relugolix 10 mg, relugolix 20 mg and relugolix 40 mg was similar to those treated with placebo. The rank (from best to worst) was: ASP1707 15 mg (0.06), ASP1707 5 mg (0.16), ASP1707 10 mg (0.31), elagolix 400 mg (0.33), ASP1707 3 mg (0.51), elagolix 150 mg (0.56), relugolix 20 mg (0.71), relugolix 10mg (0.76), and relugolix 40 mg (0.78) (Fig. 3C).

Three studies including 688 women in 10 cohorts demonstrated changes in percentage of days using analgesics from baseline. There was a significant reduction in percentage of days using analgesics in relugolix 40 mg, relugolix 20 mg and relugolix 10mg. The rank (from best to worst) was: relugolix 40 mg (0.01), relugolix 10mg (0.30), relugolix 20 mg (0.34), elagolix 250 mg (0.62), and elagolix 150 mg (0.88) (Fig. 3D).

#### Safety-related outcomes

**Fig. 4:**
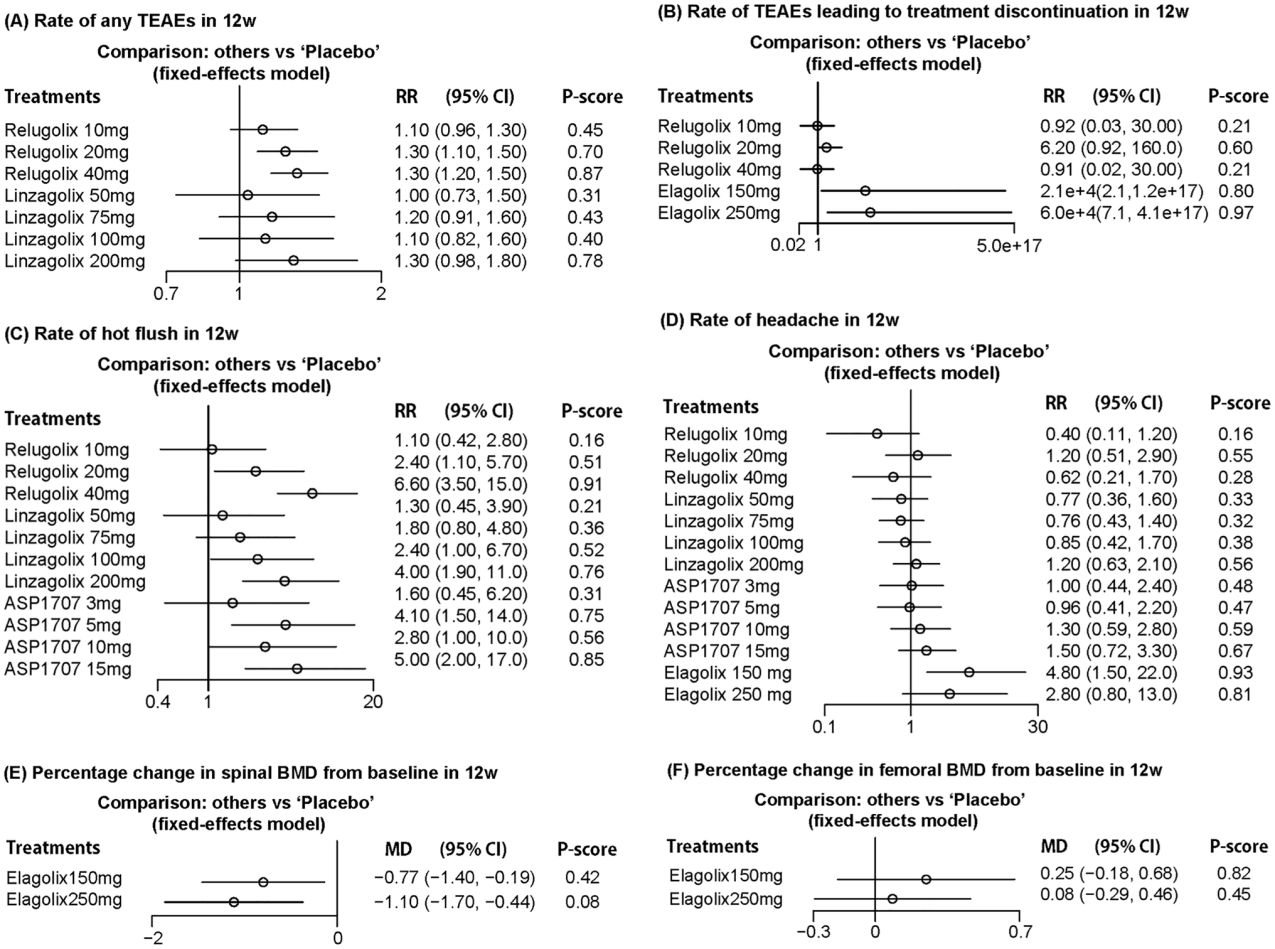
Assessment of safety related outcomes

Rate of TEAEs was reported in 2 studies including 9 cohorts and a total of 724 women. The rates of any TEAEs in patients treated with relugolix 40 mg and relugolix 20 mg were significantly higher than those treated with placebo (*RR* = 1.30, 95% CI [1.20, 1.50], *RR* = 1.30, 95% CI [1.10, 1.50]). However, the rates of TEAEs in patients receiving relugolix 10 mg, linzagolix 200 mg, linzagolix 100 mg, and linzagolix 75 mg were slightly higher than those in patients receiving placebo; while the rate of TEAEs in patients treated with linzagolix 50 mg was similar with that in patients treated with placebo. The rank of rate (from high to low) was relugolix 40 mg (0.87), linzagolix 200 mg (0.78), relugolix 20 mg (0.70), relugolix 10 mg (0.45), linzagolix 75 mg (0.43), linzagolix 100 mg (0.40), and linzagolix 50 mg (0.31) (Fig. 4A).

For rate of treatment discontinuation led by TEAEs, the data was reported by 3 studies involving 688 women in 10 cohorts. The results showed that the rates of treatment discontinuation in patients treated with elagolix 150 mg and elagolix 250 mg were remarkably higher than those receiving other treatments (*RR* = 2.1e + 4, 95% CI [2.1,1.2e + 17], *RR* = 6.0e + 4, 95% CI [7.1, 4.1e + 17]). The rank (from high to low) was: elagolix 250 mg (0.97), elagolix 150 mg (0.80), relugolix 20 mg (0.60), relugolix 40 mg (0.21), and relugolix 40 mg (0.21) (Fig. 4B).

As common adverse effect of oral non-peptide GnRH antagonists, hot flush was reported in three studies involving 1167 women in 14 cohorts. We found that relugolix 40 mg, relugolix 20 mg, linzagolix 200 mg, linzagolix 100 mg, ASP1707 15 mg, and ASP1707 5 mg increased the rate of hot flush significantly (*RR* = 6.60, 95% CI [3.50, 15.0], *RR* = 2.40, 95% CI [1.10, 5.70], *RR* = 4.00, 95% CI [1.90, 11.0], *RR* = 2.40, 95% CI [1.00, 6.70], *RR* = 5.00, 95% CI [2.00, 17.0], *RR* = 4.10, 95% CI [1.50, 14.0]). The rank (from high to low) was: relugolix 40 mg (0.91), ASP1707 15 mg (0.85), linzagolix 200 mg (0.76), ASP1707 5 mg (0.75), ASP1707 10 mg (0.56), linzagolix 100 mg (0.52), relugolix 20 mg (0.51), linzagolix 750 mg (0.36), ASP1707 3 mg (0.31), and relugolix 20 mg (0.16) (Fig. 4C).

Rate of headache was analyzed in 5 studies involving 1452 women in 20 cohorts. The highest rate of headache was found in patients receiving elagolix 150 mg (*P* score = 0.93), followed by that in patients receiving elagolix 250 mg (*P* score = 0.81) (Fig. 4D).

Furthermore, percentage changes in spinal BMD and femoral BMD were assessed in two studies with 278 women in 6 cohorts. For percentage change of spinal BMD, our results showed significant decrease in patients treated with elagolix 150 mg and elagolix 250 mg (MD =  − 0.77, 95% CI [− 1.40, − 0.19], *MD* =  − 1.10, 95% CI [− 1.70, − 0.44]). The change of spinal BMD in patients treated with elagolix 150 mg (*P* score = 0.42) was less than that in patients treated with elagolix 250 mg (*P* score = 0.08) (Fig. 4E). For percentage change in femoral BMD, no significant difference was found in patients treated with elagolix 150 mg or 250 mg comparing with those treated with placebo (Fig. 4F).

### Heterogeneity and inconsistency

There was no significant heterogeneity across studies in all quantitative analysis.

## Discussion

Oral non-peptide GnRH antagonists are novel treatment options for endometriosis-associated pain [20]. However, evidence of direct comparison among different oral non-peptide GnRH antagonists was lacking. In this NMA, we assessed 6 studies with 2732 women that compared varying types and dosage of oral non-peptide GnRH antagonists with placebo in treating moderate-to-severe endometriosis-associated pain. For pain-related outcomes, almost all oral non-peptide GnRH antagonists were effective, except for linzagolix 50 mg and ASP1707 3 mg. For safety-related outcomes, most of the oral non-peptide GnRH antagonists brought about more adverse effects than placebo.

Elagolix is the first oral GnRH antagonists approved by FDA for the management of endometriosis-associated pain [21]. In our NMA, elagolix 400 mg was the most effective in managing pelvic pain and dyspareunia. In consistent with our results, elagolix 400 mg was recommended in patients with co-existing dyspareunia [10]. We also found that lower dose of elagolix (250 mg) could ameliorate pelvic pain significantly and remarkable reduced analgesics use. Moreover, elagolix 150 mg has been proposed to long-term use in treating endometriosis-associate pain [22]. Consistently, in our study, significant reductions in pelvic pain and dyspareunia were achieved in patients treated with elagolix 150 mg, though more analgesics were used compared with placebo. Thus, the effect of analgesics on pain controlling could not be ruled out, and more evidence is needed. For safety-outcomes, both dosages (250 mg and 150 mg) of elagolix increased the incidence of headache, one of the most common adverse effects reported by previous study leading to treatment discontinuation [22], which suggested a higher probability of TEAEs when receiving elagolix treatment.

Relugolix is an oral GnRH antagonists approved by FDA in uterine fibroids in 2019 [23], and its combination tablets were approved for endometriosis in 2022. In treating endometriosis-associated dysmenorrhea, we found that the efficacy of relugolix 40 mg ranked the second, and a dose-dependent reduction of analgesics use was detected. In the safety assessment, the rate of hot flush in patients treated with relugolix 40 mg was the highest, while treatment discontinuation due to TEAEs was similar with placebo. Additionally, previous study has reported that long-term application of relugolix 40 mg was well tolerable [24]. Unexpectedly, relugolix showed little effect in treating dyspareunia, regardless of the dosage. We speculated this might be due to the small sample size and less sexual intercourse due to pain, future study with larger sample size is needed.

ASP1707, which is developed for the treating endometriosis and rheumatoid arthritis by Astellas Pharma [25], was in the leading position in relieving dysmenorrhea and dyspareunia in our analysis, which supports the potential benefit of ASP1707. Our results also showed that the efficacy and adverse effects of ASP1707 were in a dose-dependent manner. Due to limited clinical trials, the optimal strategy of ASP1707 needs to be explored further.

Linzagolix is a novel type of oral GnRH antagonist and is currently in late experimental clinical trial [11]. In managing overall pelvic pain, linzagolix 75 mg was superior to linzagolix 200 mg in this NMA. For safety outcomes, the TEAEs of linzagolix were dose-dependent, which is that the rate of TEAEs was the highest in patients receiving linzagolix 200 mg, and the rate was at a medium level without significant difference comparing with placebo in patients treated with linzagolix 75 mg. Taken together, linzagolix 75 mg might be the optimal strategy in 12w.

In the treatment of ASP1707 and linzagolix, our results showed that a higher dose sometimes gains a worse effect in terms of pain in 12w. We speculated these might be due to following reasons: Firstly, the patient-reported outcomes used to assess pain were subjective; secondly, we could only obtain data at 12w, which was not long enough to obtain obvious pain relief; moreover, studies demonstrated that with the extension of the treatment duration; the effect of pain relief showed a dose-dependent manner [12, 18]. Taken together, more objective pain assessment methods and long-term treatments need to be explored in the future.

In the present analysis, the spinal and femoral BMD of patients treated with elagolix were assessed. We found spinal BMD decreased more significantly in patients treated with elagolix 250 mg than those with elagolix 150 mg. Decreased BMD was considered to be a key factor constrained the long-term use of oral GnRH antagonists [26]. Nevertheless, a long-term study concluded that treatment with elagolix had minimal impact on BMD over a 24-week period [27]. Furthermore, our results showed that the femoral BMD in patients treated with elagolix was increased. The authors inferred that the different population enrolled might explain the increased femoral BMD partially [19]. Therefore, multicenter and multination RCTs covering different races should be implemented to determine the role of elagolix or oral GnRH antagonist on BMD.

Several limitations should be noted in our NMA. Firstly, limited number of eligible studies may constrain the confidence of our findings. Secondly, the sample size of some included studies is relatively small. Thirdly, the population was restrained to few nations, and data of long-term effects, headache before treatment and sexual activity could not be obtained. Last but not least, unified tools for outcome measurement such as pain and BMD should be adopted to obtain more objective evaluation.

## Conclusion

In the present NMA, our findings indicated that oral GnRH antagonists were effective in treating endometriosis-associated pain in 12w, and the efficacy and safety of oral GnRH antagonists were dose-dependent. Except for linzagolix 75 mg, high dose of oral GnRH antagonists was favorable. Multicenter and multination RCTs with larger sample size and variety of races were urgently needed in the future.

## Data Availability

Not applicable.
